# The effect of intraoperative transnasal humidified rapid-insufflation ventilatory exchange on emergence from general anesthesia in patients undergoing microlaryngeal surgery: a randomized controlled trial

**DOI:** 10.1186/s12871-023-02169-y

**Published:** 2023-06-13

**Authors:** Wei Wei, Xiang Li, Lili Feng, Jiali Jiao, Wenxian Li, Yirong Cai, Rui Fang, Yuan Han

**Affiliations:** 1 Department of Anesthesiology, Eye & ENT Hospital of Fudan University, Xuhui District, Shanghai, 200031 China; 2 Institute of Translational Medicine, Shanghai Jiao Tong University, Minhang District, Shanghai, China; 3 Department of Otolaryngology, Eye & ENT Hospital of Fudan University, Xuhui District, Shanghai, China

**Keywords:** THRIVE, Laryngeal mask, Tubeless anesthesia, Microlaryngeal surgery, Emergence from general anesthesia

## Abstract

**Background:**

Transnasal humidified rapid-insufflation ventilatory exchange (THRIVE) has received extensive attention for its utility in tubeless anesthesia. Still, the effects of its carbon dioxide accumulation on emergence from anesthesia have not been reported. This randomized controlled trial aimed at exploring the impact of THRIVE combined with laryngeal mask (LM) on the quality of emergence in patients undergoing microlaryngeal surgery.

**Methods:**

After research ethics board approval, 40 eligible patients receiving elective microlaryngeal vocal cord polypectomy were randomly allocated 1:1 to two groups, THRIVE + LM group: intraoperative apneic oxygenation using THRIVE followed by mechanical ventilation through a laryngeal mask in the post-anesthesia care unit (PACU), or MV + ETT group: mechanically ventilated through an endotracheal tube for both intraoperative and post-anesthesia periods. The primary outcome was duration of PACU stay. Other parameters reflecting quality of emergence and carbon dioxide accumulation were also recorded.

**Results:**

Duration of PACU stay (22.4 ± 6.4 vs. 28.9 ± 8.8 min, *p* = 0.011) was shorter in the THRIVE + LM group. The incidence of cough (2/20, 10% vs. 19/20, 95%, *P* < 0.001) was significantly lower in the THRIVE + LM group. Peripheral arterial oxygen saturation and mean arterial pressure during intraoperative and PACU stay, Quality of Recovery Item 40 total score at one day after surgery and Voice Handicap Index-10 score at seven days after surgery were of no difference between two groups.

**Conclusions:**

The THRIVE + LM strategy could accelerate emergence from anesthesia and reduce the incidence of cough without compromising oxygenation. However, these benefits did not convert to the QoR-40 and VHI-10 scores improvement*.*

**Trial registration:**

ChiCTR2000038652.

## Introduction

The most crucial consideration for anesthesia management in upper airway surgery is to maintain the patient's airway for optimal surgical exposure as well as to keep adequate ventilation and depth of anesthesia. Tubeless anesthesia provides an unobstructed surgical field, thus is more preferable in shared airway surgery. Before high-flow oxygen devices appeared, tubeless anesthesia was accomplished through either spontaneous breathing [1] or intermittent ventilation within apnea. The traditional approach of apneic oxygenation can lead to severe hypoxemia combined with hypercapnia, which induces severe complications. Transnasal humidified rapid-insufflation ventilatory exchange (THRIVE) is a new technique of apneic oxygenation [2, 3]. Several exploratory studies have suggested that THRIVE could be used for apneic oxygenation during anesthesia induction and difficult airway management [3–10]. However, due to the insufficient carbon dioxide exchange capacity, the safety of applying THRIVE to patients without spontaneous respiration needs further evaluation.

Theoretically, THRIVE could facilitate carbon dioxide clearance through mechanisms such as dead space flushing and cardiogenic oscillations [11]. However, previous study showed that carbon dioxide accumulation was an inevitable adverse effect of THRIVE [12]. In patients receiving THRIVE, partial pressure of carbon dioxide in arterial blood (PaCO_2_) increased linearly with the duration of apnea regardless of the flow rate used [12]. Hypercapnia affects the oxygen-carrying capacity of the blood, the circulatory system, spontaneous breathing, acid–base balance, and the nervous system [13]. Mild to moderate hypercapnia increases cardiac output, reduces vascular resistance, and increases oxygen supply to tissues [14–16]. Hypercapnia is considered organ-protective compared to hypocapnia [17], and in some cases, it is well tolerated in anesthetized patient [18]. Hypercapnia may lead to hemodynamic instability and may influence emergence from anesthesia [19–24]. The current evidence showed that compared with mechanical ventilation through an endotracheal tube, using THRIVE in short duration surgery was not associated with obvious intraoperative adverse events [3, 25, 26]. However, research regarding the impact of THRIVE on the quality of emergence is still lacking. In addition, the options of ventilation strategy after THRIVE were varied, such as ventilation through an endotracheal tube (ETT) or a laryngeal mask (LM), and spontaneous respiration. However, there was no clear evidence of which option was better.

To explore the effects of intraoperative THRIVE followed by mechanical ventilation through LM for post-anesthesia care on the quality of emergence from general anesthesia in patients undergoing shared airway surgery such as laryngoscopy, we designed this single-center randomized controlled trial. The primary outcome was the duration of post-anesthesia care unit (PACU) stay, and the secondary outcomes were the time to spontaneous respiration recovery, cough in PACU, and changes in perioperative vital signs.

## Methods

This study was approved by the Ethics Committee of Eye & ENT Hospital of Fudan University (2018033–1) and registered at https://www.chictr.org.cn on 27/09/2020 (ChiCTR2000038652). This randomized, controlled, patient and data analysts blinded study enrolled adult patients scheduled for elective microlaryngeal vocal cord polypectomy at Eye & ENT Hospital of Fudan University (Shanghai, China); all patients provided written informed consent.

### Patient inclusion and exclusion criteria

All patients were aged 18–60 years, had an ASA physical status 1–2, and were scheduled for elective microlaryngeal vocal cord polypectomy with anticipated surgery time less than 20 min. Exclusion criteria are: severe upper airway obstruction, known difficult mask ventilation or difficult intubation, skull base fracture or other defects, severe cardiovascular or cerebrovascular diseases, upper respiratory tract infection within two weeks, bronchiectasis or other pulmonary diseases, neuromuscular disorders, obesity with body mass index (BMI) above 35, severe or poorly controlled gastroesophageal reflux disease, pregnancy, allergy to any agent used in the study.

### Randomization

An independent research coordinator used SPSS (Version 23.0) to make randomized number sequence and prepared sealed and opaque envelopes according to the number sequence. Anesthesiologists assigned eligible patients using these envelopes before anesthesia induction. Patients were randomly assigned to either intraoperative THRIVE followed by mechanical ventilation through a LM for post-anesthesia care (THRIVE + LM) group or mechanical ventilation through an endotracheal tube for both intraoperative and post-anesthesia care periods (MV + ETT) group. The allocation ratio was 1:1. The randomization schedule was stratified into blocks of 4. All randomized patients received the protocolized study intervention according to their allocation. Patients, investigators who interview patients for QoR-40 and VHI, and data analysts are blinded to group allocation. Since anesthesiologists, otolaryngologists, and post-anesthesia care providers (outcome assessors) cannot be blinded due to the obvious difference in ventilation strategies between the THRIVE + LM group and the MV + ETT group, none of them are invited as investigators of this study, so they are not interested parties.

### Anesthesia management

All patients received routine preoperative preparation with no premedication. Standard intraoperative monitoring, including five lead electrocardiography, pulse oximetry, non-invasive blood pressure, bispectral index, and transcutaneous carbon dioxide (Sentec®, SenTec AG, Terwill, Switzerland), was applied to all patients. Pre-oxygenation was performed via a facemask using pure oxygen at a flow of 10 L/min until fractional expired oxygen ≥ 90%. Anesthesia induction consisted of intravenous target-controlled infusion of propofol at an effect-site concentration of 4 μg/ml and remifentanil at 5.0 ng/ml, and intravenous bolus of fentanyl 1 μg/kg for analgesia, succinylcholine 2 mg/kg for neuromuscular blockade. For anesthesia maintenance, the propofol target was adjusted to maintain BIS between 40 and 50, and the remifentanil target was adjusted to maintain mean arterial pressure (MAP) ranging between 80 and 120% of the preoperative MAP. Additional 0.5 mg/kg of succinylcholine was added every five minutes until the end of surgery. Intravenous ephedrine 6 mg was given to rescue hypotension (MAP < 65 mmHg), and intravenous atropine 0.5 mg was given to rescue bradycardia (heart rate, HR < 60 bpm). Palonosetron 0.25 mg and dexamethasone 5 mg were given intravenously to prevent postoperative nausea and vomiting.

Patients were intubated with an ID 6 mm reinforced endotracheal tube using a video laryngoscope in the MV + ETT group. Patients were ventilated under volume-controlled ventilation mode, tidal volume of 8 ml/kg, respiratory rate of 12 bpm, the inspiration-expiration ratio of 1:2, oxygen concentration of 50%, oxygen flow of 2 L/min. Ventilator parameters were adjusted to maintain the end-tidal carbon dioxide pressure (P_ET_CO_2_) between 35 and 45 mmHg. In the THRIVE + LM group, the optiflow® (Fischer & Paykel Healthcare, Auckland, New Zealand) was turned on in advance: temperature 37 ℃, flow rate 60 L/min, oxygen concentration 100%. A nasal catheter of proper size was put on the patient's forehead. After anesthesia induction, the nasal catheter was inserted into the patient’s nostrils, and apneic oxygenation began. Upper airway patency was kept by jaw thrust before the surgical laryngoscope was suspended. THRIVE discontinuation criteria are: 1. apnea lasting more than 20 min, 2. transcutaneous carbon dioxide monitoring exceeding 80 mmHg, and 3. severe adverse events such as malignant arrhythmia. Once the discontinuation criteria were triggered, THRIVE was terminated, tracheal intubation or laryngeal mask placement and mechanical ventilation were performed, and the procedure was subsequently completed according to the conventional protocol. At the end of the surgery, a Size 4 LMA® Supreme was inserted for mechanical ventilation after the surgical laryngoscope was withdrawn. The ventilator parameters were identical to those in the MV + ETT group. All patients were transported to PACU after the end of surgery. After arriving at PACU, patients received monitoring including five lead electrocardiography, pulse oximetry, and non-invasive blood pressure. Mechanical ventilation was conducted under pressure-targeted synchronized intermittent mandatory ventilation mode, with an oxygen concentration of 50%, and safety backup parameters consisting of tidal volume of 7 to 8 ml/kg, respiratory rate of 10 to 12 bpm, and the inspiration-expiration ratio of 1:2.

During the intraoperative period, the heart rate (HR), MAP, peripheral arterial oxygen saturation (SpO_2_), transcutaneous carbon dioxide pressure (P_tc_CO_2_) were recorded before anesthesia induction (baseline), after anesthesia induction, and every 2 min after the start of surgery until 10 min. During the postoperative period the HR, MAP, SpO_2_, P_ET_CO_2_ were recorded at the end of surgery and every 5 min after the arrival at PACU until 30 min. At the end of the surgery, an arterial blood gas analysis was performed in all patients. The Quality of Recovery item 40 (QoR-40) scale was tested 1 day before and 1 day after surgery, and Voice Handicap Index-10 (VHI-10) scale were tested 1 day before and 7 days after surgery.

### Outcomes

The primary outcome was the duration of PACU stay. The time was counted from the end of surgery until patients met the discharge criteria of PACU. The Modified Aldrete Score was used as PACU discharge criteria. Patient’s activity level, respiration, circulation, consciousness and oxygen saturation were evaluated. The patient must have a score of “9” to be discharged from PACU unless otherwise approved by the Anesthesiologist. The secondary outcomes included the time from the end of surgery to spontaneous respiration recovery and LM/ETT removal, anesthesia induction time, anesthesia induction and maintenance time, time from anesthesia induction to PACU discharge, intraoperative P_tc_CO_2_, postoperative P_ET_CO_2_, the results of arterial blood gas analysis, perioperative HR, MAP and SpO_2_, QoR-40 and VHI-10 score.

### Sample size and statistical analysis

Based on our previous clinical data, the average duration of PACU stay in patients undergoing microlaryngeal vocal cord polypectomy was about 30 min. The sample size of this trial was calculated based on the assumption that the difference in duration of PACU stay between the THRIVE + LM group and MV + ETT group was more than 10 min. Finally, at least 38 patients (19 in each group) were needed to verify the difference between the two groups, with α = 0.05, power = 0.80, and a drop-out rate of 10%.

Normality for continuous variables was determined using the Shapiro–Wilk test. Normal variables were presented as mean ± SD, and non-normal ones were presented as median (Interquartile Range, IQR). Student’s t-test or Mann–Whitney U test was selected to compare continuous variables, depending on the normality. χ^2^ test or Fisher’s exact test was chosen for categorical variables. For other secondary outcomes, including QoR-40 total score (score range: 40–200) and VHI-10 (score range: 0–40), the Wilcoxon rank-sum test was used to compare the difference between the THRIVE + LM group and MV + ETT group.

## Results

**Fig. 1:**
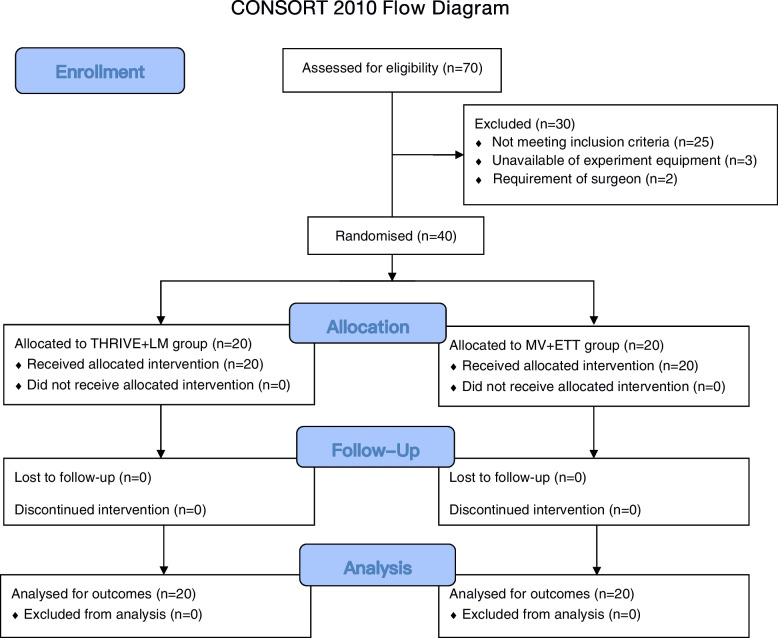
Consort Flow Diagram. *ETT* endotracheal tube, *LM* laryngeal mask, *MV* mechanical ventilation *THRIVE* transnasal humidified rapid insufflation ventilatory exchange

Between September 2020 to November 2020, a total of 70 patients were screened for eligibility, and 45 patients met eligibility criteria. Five patients were excluded because of the unavailability of the experiment equipment, or the requirement of the surgeon. Forty patients were randomized, and all patients completed the trial for the primary outcome (Fig. 1). Baseline demographic and clinical characteristics were similar between the two groups (Table 1).

**Table 1:** Baseline characteristics of 40 patients treated with THRIVE + LM or MV + ETT for microlaryngeal vocal cord polypectomy. Values are mean ± SD, or number (percentage)

	THRIVE + LM (*n* = 20)	MV + ETT (*n* = 20)
Age (y)	47.3 ± 9.1	43.5 ± 8.8
Sex (male)	7 (35%)	6 (30%)
Height (cm)	168.5 ± 8.1	167.6 ± 5.5
Weight (kg)	70.3 ± 9.3	68.6 ± 11.4
BMI (kg.m^−2)^	24.7 ± 2.2	24.3 ± 2.9
ASA physical status		
1	16 (80%)	15 (75%)
2	4 (20%)	5 (25%)
Location of vocal cord polyp		
Front	2 (10%)	5 (25%)
Middle	5 (25%)	6 (30%)
Back	1 (5%)	0 (0%)
Multiple	12 (60%)	9 (45%)

*ASA* American Society of Anesthesiologists, *BMI* body mass index, *ETT* endotracheal tube, *LM* laryngeal mask, *MV* mechanical ventilation, *THRIVE* transnasal humidified rapid insufflation ventilatory exchange

Compared with the MV + ETT group, patients in the THRIVE + LM group were discharged from PACU faster (THRIVE + LM vs. MV + ETT, 22.4 ± 6.4 vs. 28.9 ± 8.8 min, *p* = 0.011, Table 2). Spontaneous respiration recovery time, LM/ETT removal time, anesthesia induction time, anesthesia induction and maintenance time, time from anesthesia induction to PACU discharge were also shorter in the THRIVE + LM group (*p* < 0.05).

**Table 2:** Post-anesthesia characteristics of 40 patients treated with THRIVE + LM or MV + ETT for microlaryngeal vocal cord polypectomy

	THRIVE + LM (*n* = 20)	MV + ETT (*n* = 20)	*P*
Primary outcome			
Time in PACU; min	22.4 ± 6.4	28.9 ± 8.8	0.011
Secondary outcome			
Time from arriving PACU			
To spontaneous respiration; min	13.3 ± 6.5	21.0 ± 10.5	0.008
To airway tool removal; min	14.9 ± 7.2	21.5 ± 10.3	0.026
Time for anesthesia induction; min	3.4 ± 0.8	4.8 ± 0.9	< 0.001
Time for anesthesia induction and maintenance; min	11.7 ± 3.6	14.7 ± 4.5	0.024
Time from anesthesia induction to PACU discharge; min	34.2 ± 8.0	43.6 ± 10.9	0.003
P_ET_CO_2_			
End of surgery	60.0 ± 9.9	35.9 ± 3.1	< 0.001
Spontaneous respiration recovery	49 (40–55)	39 (38–42)	< 0.001
Airway tool removal	45.5 ± 9.0	38.1 ± 4.9	< 0.001
Cough in PACU; n (%)	2 (10)	19 (95)	< 0.001
QoR-40 score			
1 day before surgery	194 (193–196)	195 (194–198)	0.692
1 day after surgery	198 (197–199)	198 (194–199)	0.096
VHI-10 score			
1 day before surgery	9.5 (4.8–15.3)	6.5 (2–11.8)	0.136
7 days after surgery	3 (1–8)	2.5 (1–5)	0.594

Values are presented as mean ± SD, number (proportion), or median (IQR)

*ETT* Endotracheal tube, *LM* Laryngeal mask, *MV* Mechanical ventilation, *PACU* Post-anesthesia care unit, *PaO*_*2*_ Partial pressure of oxygen in arterial blood, *PaCO*_*2*_ Partial pressure of carbon dioxide in arterial blood, *P*_*ET*_*CO*_*2*_ End-tidal carbon dioxide pressure, *QoR-40* Quality of Recovery-40, *THRIVE* Transnasal humidified rapid insufflation ventilatory exchange, *VHI-10* Voice Handicap Index-10

**Fig. 2:**
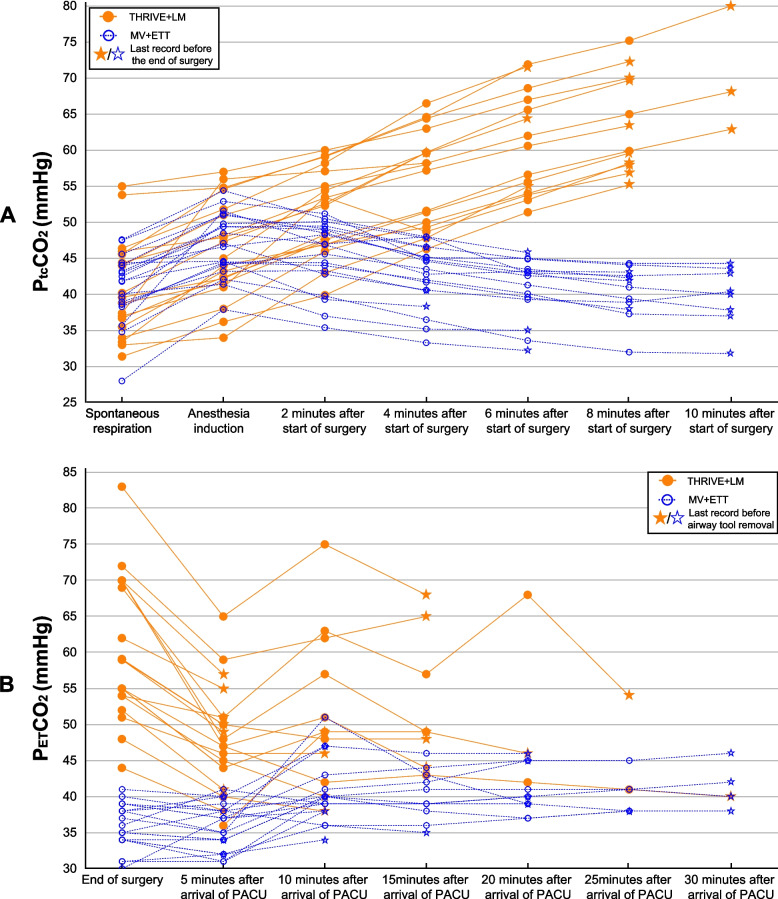
Scatter chart of **A** intraoperative P_tc_CO_2_ and **B** postoperative P_ET_CO_2_. Each line represents a single patient. THRIVE + LM group use orange solid circles and solid lines, while MV + ETT group use blue hollow circles and dotted lines. Five-pointed star represents the last record of the patient. During the intraoperative period, the P_tc_CO_2_ values increase with time of apneic oxygenation in the THRIVE + LM group. The P_tc_CO_2_ values are significantly higher in the THRIVE + LM group at 2, 4, 6, 8,10 min after the start of surgery. During the postoperative period, the P_ET_CO_2_ values are significantly higher in the THRIVE + LM group at the end of the surgery, 5 and 10 min after the arrival of PACU. *ETT* endotracheal tube, *LM* laryngeal mask, *MV* mechanical ventilation, *PACU* post-anesthesia care unit, *P*_*ET*_*CO*_*2*_ end-tidal carbon dioxide pressure, *P*_*tc*_*CO*_*2*_ transcutaneous carbon dioxide pressure, *THRIVE* transnasal humidified rapid insufflation ventilatory exchange

During the intraoperative period, the P_tc_CO_2_ increased gradually with time in the THRIVE + LM group while remained stable in the MV + ETT group (Fig. 2). While in PACU, the P_ET_CO_2_ was higher in the THRIVE + LM group than the MV + ETT group at the end of the surgery, 5 and 10 min in the PACU. From 15 min in the PACU to LM/ETT removal, the P_ET_CO_2_ was similar between the two groups.

SpO_2_ and MAP were similar between the two groups during intraoperative and PACU periods. During anesthesia induction, HR of patients in the THRIVE + LM group was significantly lower than the MV + ETT group. However, at 6, 8, and 10 min after the start of surgery, HR was higher in the THRIVE + LM group (Table 3).

**Table 3:** Other vital signs including SpO_2_, HR and MAP during general anesthesia and PACU

	THRIVE + LM (*n* = 20)	MV + ETT (*n* = 20)	*P*
SpO_2_; %			
Spontaneous respiration	99.4 ± 0.9	99.4 ± 0.8	0.998
Anesthesia induction	100.0 ± 0.2	99.8 ± 0.7	0.368
End of surgery	99.2 ± 1.8	99.7 ± 0.7	0.253
PACU Discharge	98.5 ± 1.8	98.9 ± 2.1	0.527
HR; bpm			
Spontaneous respiration	66.5 ± 9.9	72.3 ± 14.7	0.149
Anesthesia induction	61.8 ± 9.4	72.6 ± 12.6	0.004
End of surgery	73.9 ± 9.9	66.0 ± 9.4	0.014
PACU Discharge	73.8 ± 10.7	72.5 ± 9.3	0.677
MAP; mmHg			
Spontaneous respiration	96.7 ± 12.4	95.5 ± 12.9	0.755
Anesthesia induction	73.3 ± 13.8	72.4 ± 12.4	0.828
End of surgery	74.6 ± 12.5	75.1 ± 14.3	0.897
PACU Discharge	93.9 ± 11.9	94.6 ± 15.0	0.877

Values are presented as mean ± SD

*ETT* Endotracheal tube, *HR* Heart rate, *LM* Laryngeal mask, *MAP* Mean arterial pressure, *MV* Mechanical ventilation, *PACU* Post-anesthesia care unit, *SpO*_*2*_ Pulse oxygen saturation, *THRIVE* Transnasal humidified rapid insufflation ventilatory exchange

**Fig. 3:**
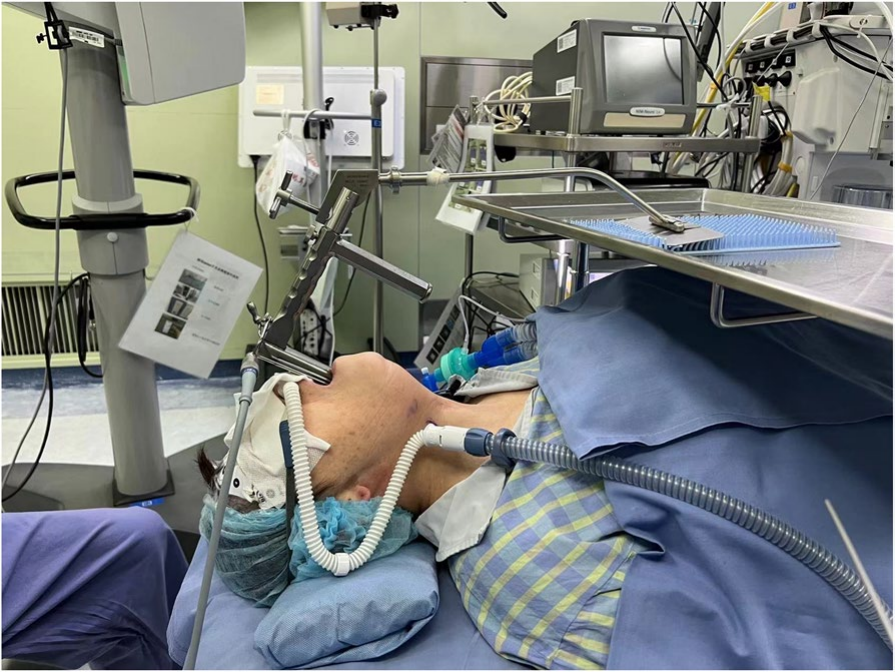
A photo of THRIVE operated in the study

Surgery duration was (THRIVE + LM vs. MV + ETT, 497.5 ± 210.4 vs. 595.7 ± 259.3 s, *p* = 0.197) similar between two groups. The arterial blood gas analysis at the end of surgery revealed that the partial pressure of oxygen in arterial blood (PaO_2_) was similar between two groups (THRIVE + LM vs. MV + ETT, 275 ± 141 vs. 293 ± 141 mmHg, *p* = 0.677). In contrast, the partial pressure of carbon dioxide in arterial blood (PaCO_2_) was significantly higher in the THRIVE + LM group (THRIVE + LM vs. MV + ETT, 77.1 ± 12.0 vs. 41.2 ± 4.1 mmHg, *p* < 0.001), as well as pH significantly lower in the THRIVE + LM group (THRIVE + LM vs. MV + ETT, 7.23 ± 0.04 vs 7.42 ± 0.04, *p* < 0.001). Figure 3 is a photo of THRIVE setting.

In the PACU, the observed cough incidence was lower in the THRIVE + LM group than in the MV + ETT group (THRIVE + LM vs. MV + ETT, 2/20, 10% vs. 19/20, 95%, *P* < 0.001). The QoR-40 score at one day after surgery, and VHI-10 score at seven days after surgery were comparable between the two groups (Table 2).

## Discussion

THRIVE could maintain oxygenation without blocking the view of surgical field, especially when operating lesions at the middle and lower part of vocal cords. However, the intraoperative carbon dioxide accumulation may affect the quality of emergence from general anesthesia in patients using THRIVE. LM is less irritating to the airway but not as suitable as the endotracheal tube for microlaryngeal surgery. Therefore, the potential influence of THRIVE combined with LM on the quality of emergence from anesthesia is worthy of study. Our study revealed that compared with the conventional mechanical ventilation through an endotracheal tube, patients treated with the THRIVE + LM strategy: 1. recovered spontaneous respiration, met airway tool removal and PACU discharge criteria faster; 2. had a lower incidence of cough in the PACU; 3. could maintain adequate oxygenation in a short operative time; 4. have apnea-duration related intraoperative carbon dioxide accumulation.

Although THRIVE is theoretically capable of facilitating carbon dioxide clearance [11], our study found that patients in the THRIVE + LM group experienced carbon dioxide accumulation, manifested as elevated P_tc_CO_2_ increasing linearly with the progress of apnea. Booth et al. studied tubeless anesthesia using high flow nasal oxygen: they compared apneic oxygenation with spontaneous breathing and found that after 30 min’ apneic oxygenation, PaCO_2_ was 89.0 ± 16.5 mmHg in apneic oxygenation group vs 55.2 ± 7.2 mmHg in spontaneous breathing group [27]. Generally, permissive hypercapnia for ARDS treatment allows PaCO_2_ values of 45 ~ 55 mmHg with pH above 7.25 [28]. THRIVE resulted in much higher level of hypercapnia, and this is the most important concern in THRIVE technique. Hypercapnia led to a hyperdynamic state of the circulatory system with an increase of HR and MAP. This finding was consistent with previous studies [12]. Likewise, our study did not find other adverse events related to carbon dioxide accumulation. However, this conclusion was based on non-obese patients with healthy lungs, and iatrogenic severe hypercapnia should always be avoided whenever possible. For patients with comorbidities such as obesity and chronic obstructive pulmonary disease, THRIVE may lead to severe hypercapnia. Thus, further studies are necessary to prove the safety of THRIVE in these specific patients.

After arriving at PACU, patients in the THRIVE + LM group recovered spontaneous respiration, get LM removed, and met PACU discharge criteria faster than patients in the MV + ETT group. This finding has not been reported in previous studies. We found out that the difference in duration of PACU stay between the two groups mainly derived from the shorter time of spontaneous respiration recovery, suggesting the stimulation of the respiratory center by hypercapnia may be the crucial mechanism. Carbon dioxide is an agonist of respiration, and hypercapnia at 100–150 mmHg has the most substantial agonistic effect on spontaneous respiration but is highly correlated with anesthetics used. Healthy volunteer study showed that hypercapnia-induced ventilation increase is associated with bilateral changes in activity of anterior cingulate cortex [29]. The main objective of this study is to observe the effects THRIVE has on patients’ emergence quality and post-operative recovery, but interestingly, THRIVE brought incidental benefits of faster spontaneous breathing recovery and awakening by coincidence. This might have some positive effect on faster patients turnover and more efficient use of healthcare resources depending on specific context. In our study, the PaCO_2_ was not analyzed at PACU discharge. One previous study proved that the PaCO_2_ reduced from over 80 mmHg at the end of surgery to about 40 mmHg at the discharge from PACU [27]. This phenomenon indicates that hypercapnia caused by short-term use of THRIVE is correctable before discharging from the PACU.

Safaeian found that compared with an endotracheal tube, laryngeal mask reduced the incidence of postoperative adverse events such as hoarseness, cough, and shortness of breath in prolonged ENT surgeries such as functional endoscopic sinus surgery, parotid surgeries, rhinoplasty, and septoplasty, but patients with surgeries involving the throat or other lower part of the airway was excluded from the study [30]. We observed that mechanical ventilation through a LM following the THRIVE was not associated with severe respiratory adverse events, indicating that LM is feasible for post-anesthesia care in patients undergoing microlaryngeal surgery. In addition, compared with the endotracheal tube, the LM is associated with lower stress levels before and after airway tool removal [31]. In this study, we found LM could reduce the incidence of cough in the PACU and further improves the quality of emergence from anesthesia. Although aspiration in airway management with the laryngeal mask is uncommon, careful selection of patients and surgical approaches are critical [32]. It is worth noting that for patients at risk of surgical wound bleeding, using a LM instead of an endotracheal tube for airway management may lead to aspiration, so the anesthesiologist should confirm the risk of bleeding with the surgeon before deciding the ventilation strategy following THRIVE.

After 10 min of apneic oxygenation, the median PaO_2_ in the THRIVE + LM group was 264 mmHg, comparable to that in the MV + ETT group. In one previous study, Anton et al. revealed that the PaO_2_ was 259 mmHg in patients after 30 min of apneic oxygenation using THRIVE [27]. These results demonstrated that THRIVE could maintain intraoperative oxygenation for short surgery. However, this conclusion is also based on non-obese patients with healthy lungs.

This study has the following limitations. Firstly, like most previous studies, this study selected non-obese patients with healthy lungs as the study population for safety reasons. Therefore, the effects of the THRIVE + LM strategy need further study in patients with impaired pulmonary function. Secondly, the sample size of this study was relatively small. However, since the expected primary outcome is consistent with the actual one, the sample size is sufficient. Notably, the small sample size may result in a lack of data on serious adverse events, such as wound bleeding and the following aspiration. Thirdly, for practical reasons, the P_tc_CO_2_ data in the PACU was not collected, resulting in inconsistent records of intraoperative and postoperative carbon dioxide accumulation. This incoherence may lead to inaccurate perceptions of changes in carbon dioxide accumulation within the PACU but has a limited impact on the between-group comparisons. Fourthly, a repeated dose of succinylcholine might have some potential adverse effects, such as hyperkalemia, trismus, and bradycardia. If possible, rocuronium plus sugammadex would be a better choice.

## Conclusions

Compared with mechanical ventilation through an endotracheal tube, the THRIVE + LM ventilation strategy in patients undergoing microlaryngeal vocal cord polypectomy could accelerate the spontaneous respiration recovery, airway tool removal, and PACU discharge without compromising oxygenation. Furthermore, the incidence of cough decreased significantly under THRIVE + LM strategy. However, these benefits did not convert to the QoR-40 and VHI-10 score improvements.

## Data Availability

The datasets generated and analyzed during the current study are not publicly available due to institutional restrictions but are available from the corresponding author on reasonable request.

## Abbreviations

ASA: American Society of Anesthesiologists
BMI: Body mass index
ETT: Endotracheal tube
HR: Heart rate
LM: Laryngeal mask
MAP: Mean arterial pressure
MV: Mechanical ventilation
PaCO_2_: Partial pressure of carbon dioxide in arterial blood
PACU: Post-anesthesia care unit
P_ET_CO_2_: End-tidal carbon dioxide pressure
P_tc_CO_2_: Transcutaneous carbon dioxide pressure
QoR-40: Quality of recovery item 40
SpO_2_: Pulse oxygen saturation
THRIVE: Transnasal humidified rapid-insufflation ventilatory exchange
VHI-10: Voice handicap index-10
